# Harnessing DNA Double-Strand Break Repair for Cancer Treatment

**DOI:** 10.3389/fonc.2019.01388

**Published:** 2019-12-10

**Authors:** Anika Trenner, Alessandro A. Sartori

**Affiliations:** Institute of Molecular Cancer Research, University of Zurich, Zurich, Switzerland

**Keywords:** DSB repair, homologous recombination (HR), BRCA, alternative end joining (a-EJ), PARP inhibition (PARPi), DNA polymerase theta, synthetic lethality, cancer therapy

## Abstract

DNA double-strand breaks (DSBs) are highly deleterious, with a single unrepaired DSB being sufficient to trigger cell death. Compared to healthy cells, cancer cells have a higher DSB burden due to oncogene-induced replication stress and acquired defects in DNA damage response (DDR) mechanisms. Consequently, hyperproliferating cancer cells rely on efficient DSB repair for their survival. Moreover, augmented DSB repair capacity is a major cause of radio- and chemoresistance and, ultimately, cancer recurrence. Although inherited DDR defects can predispose individuals to develop certain cancers, the very same vulnerability may be therapeutically exploited to preferentially kill tumor cells. A paradigm for DNA repair targeted therapy has emerged in cancers that exhibit mutations in *BRCA1* or *BRCA2* tumor suppressor genes, conferring a strong defect in homologous recombination, a major and error-free DSB repair pathway. Clinical validation of such approaches, commonly described as synthetic lethality (SL), has been provided by the regulatory approval of poly(ADP-ribose) polymerase 1 inhibitors (PARPi) as monotherapy for *BRCA1/2*-mutated breast and ovarian tumors. In this review, we will describe the different DSB repair mechanisms and discuss how their specific features could be exploited for cancer therapy. A major emphasis is put on advances in combinatorial treatment modalities and SL approaches arising from DSB repair pathway interdependencies.

## Introduction

The integrity of our genome is constantly challenged by endogenous and exogenous insults that can induce DNA damage. To counteract genotoxic threats, cells are equipped with a diverse set of DNA damage signaling and repair mechanisms, collectively known as the DNA damage response (DDR) (1). During tumorigenesis, however, precancerous cells frequently acquire loss-of-function alterations in DDR genes, including core components of selected DNA repair pathways, to accelerate mutagenesis and become malignant (2). While healthy cells have to deal with a minor amount of damage and take advantage of the full DNA repair capacity, malignant cells are frequently equipped with reduced DNA repair functionality to cope with increased replication stress and elevated levels of endogenous DNA damage (3). Consequently, cancer cells become even more dependent on DNA repair mechanisms to survive and proliferate. Conventional treatment modalities such as radiation therapy and certain forms of chemotherapy have been built on the premise to force DNA damage-induced cell death. In summary, cancer cells are often compromised in their ability to adequately process DNA damage, which exerts selective pressure to sustain DNA repair through upregulation of mutagenic pathways, ultimately promoting disease progression and therapy resistance (4, 5).

**Figure 1:**
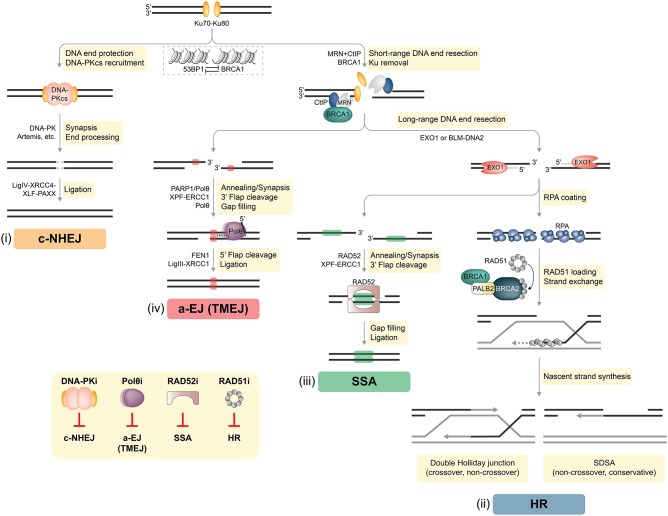
DSB repair pathways in mammalian cells. Two-ended DSBs are preferably repaired by two major competing pathways: classical non-homologous end joining (c-NHEJ) and homologous recombination (HR). In addition, DSBs can be subjected to alternative end joining [a-EJ, also referred to as DNA polymerase theta-mediated end joining (TMEJ)] or single-strand annealing (SSA). BRCA1 and 53BP1 are placed at the center of DSB repair pathway choice. Whereas, chromatin recruitment of 53BP1 drives c-NHEJ, BRCA1 antagonizes 53BP1 to channel DSB repair into HR. **(i)** C-NHEJ begins with Ku70-Ku80 (Ku) binding to DSB ends, followed by the recruitment of DNA-dependent protein kinase catalytic subunit (DNA-PKcs), forming the DNA-PK holoenzyme implicated in DNA synapsis. If necessary, DNA-PK coordinates limited processing of incompatible or chemically modified DNA ends by nucleases (e.g., Artemis) and other enzymes. The DNA ligase IV (LigIV)-XRCC4-XLF-PAXX complex executes the final ligation step. DNA end resection interferes with the default engagement of c-NHEJ by removing Ku from DNA ends, which is a critical step for initiating HR **(ii)**. First, the MRE11-RAD50-NBS1 (MRN) complex senses the DSB and with the help of BRCA1 and CtIP promotes limited resection of the 5′ strand. Next, more extensive 5′-3′ resection by exonuclease 1 (EXO1), or by the Bloom's syndrome (BLM) helicase together with the DNA2 nuclease, generates long 3′ ssDNA overhangs that become rapidly coated with the RPA heterotrimer. The BRCA1-PALB2-BRCA2 complex disassembles RAD51 heptamers and loads monomeric RAD51 onto ssDNA, promoting RAD51 filament assembly. Template-dependent strand extension is followed by “synthesis-dependent strand annealing” (SDSA), resulting in a non-crossover gene conversion. Alternatively, capture of the second ssDNA by the D-loop forms a double Holliday junction intermediate, which can be resolved either as a non-crossover or as a crossover. **(iii)** SSA requires at least 20–25 base pairs (bp) of DNA sequence homology, which are typically found between repetitive elements (indicated as green boxes) in the genome. Subsequently, RAD52 promotes annealing of complementary ssDNA and leftover non-homologous flaps of the 3′ overhangs are cleaved by XPF-ERCC1. The factors that promote gap filling and ligation during SSA remain largely elusive. **(iv)** In contrast to SSA, a-EJ (or TMEJ) utilizes short microhomologies (MHs) of 2–20 bp (indicated as red boxes) to join the two DNA strands. PARP1 has been implicated in promoting DNA end synapsis and recruiting the specialized DNA polymerase θ (Polθ) to DSBs. Polθ stabilizes MH-mediated joints between the two DNA ends serving as primers for fill-in synthesis. 3′ flaps extending from the joints are removed by XPF/ERCC1. Flap endonuclease 1 (FEN1) has recently been implicated in the removal of 5′ flaps generated by Polθ-mediated strand displacement, while the DNA Ligase III (LigIII)-XRCC1 complex is essential for the final ligation step. *Inset, bottom left:* DSB repair pathway-specific inhibitors. Inhibition of c-NHEJ has so far been mainly achieved by targeting DNA-PK using different small molecule inhibitors. Strategies to inhibit a-EJ and SSA focus on targeting their respective DNA annealing factors Polθ and RAD52, while the primary target to disrupt HR is RAD51 (see text for more details).

DNA double-strand breaks (DSBs) are considered the most lethal of all DNA lesions, eliciting the majority of the cytotoxic effects induced by ionizing radiation (IR) and certain anti-cancer drugs. Therefore, DSB repair represents a potent and targetable vulnerability in cancer cells. In healthy somatic cells two-ended DSBs are mainly repaired by two pathways: classical non-homologous end joining (c-NHEJ) and homologous recombination (HR) (Figure 1). Auxiliary mechanisms of DSB repair include single-strand annealing (SSA) and alternative end joining (a-EJ) that rely on the presence of larger repeat sequences and microhomologies at the breakpoint, respectively [(6, 7); Figure 1]. Importantly, functional interdependencies between different DNA repair pathways and within compensatory DSB repair mechanisms offer therapeutic opportunities to selectively treat DDR-deficient tumors based on the concept of synthetic lethality (SL) (3, 5, 8, 9).

## DSB Repair Pathways

The decision as to whether a given DSB is processed by c-NHEJ, HR, or alternative repair pathways is determined by several factors, including genetic and genomic background, DSB complexity, chromatin state, and cell cycle phase. For instance, c-NHEJ operates throughout the cell cycle, whereas HR relies on the presence of an undamaged sister chromatid and is therefore restricted to late S/G2 (7, 10). Therefore, HR activation requires high cyclin-dependent kinase (CDK) activity (11). In addition, numerous HR genes are found upregulated in S/G2 phase of the cell cycle (7). At the chromatin level, the appropriate equilibrium between HR and c-NHEJ is mainly established by BRCA1 and 53BP1, large DDR adaptor proteins that are enriched at DSB sites (12, 13). Whereas, 53BP1 mediates c-NHEJ events and is pivotal in repairing programmed DSBs (e.g., during class-switch recombination), BRCA1 antagonizes 53BP1 to promote DSB resection and HR [(14, 15); Figure 1]. Importantly, one-ended DSBs, predominantly induced by fork breakage or collapse due to high replication stress, lack an adjacent second DNA end for rejoining and can only be repaired by HR-related mechanisms (7).

### C-NHEJ

C-NHEJ is accountable for the repair of most two-ended DSBs in mammalian cells (Figure 1). Rapid and high-affinity binding of the Ku70-Ku80 heterodimer (Ku) to DNA ends is followed by the recruitment of DNA-dependent protein kinase catalytic subunit (DNA-PKcs), forming the active DNA-PK holoenzyme. Key functions of DNA-PK in c-NHEJ are (i) promoting synapsis of the broken ends, (ii) coordinating necessary processing of incompatible ends by DNA nucleases (e.g., Artemis) and polymerases, and (iii) engaging the DNA ligase complex composed of DNA ligase IV, XRCC4, XLF, and PAXX (7, 16). Despite rejoining DSBs without the use of extensive sequence homology, c-NHEJ is often highly accurate and its core factors therefore considered as genome “caretakers” (10, 17, 18).

### HR

In case c-NHEJ fails or is inappropriate, DSBs are subjected to extensive 5′-end resection, generating 3′-single-stranded (ss) DNA overhangs that interfere with Ku loading and promote high-fidelity repair by HR [(7, 19); Figure 1]. In a first step, the MRE11-RAD50-NBS1 (MRN) complex in conjunction with CtIP, also known as RBBP8, coordinates tethering and short-range nucleolytic degradation of DSB ends (20, 21). MRE11 exhibits a dual endo- and exonuclease activity that is critical for DNA end resection (22). Following long-range resection carried out by EXO1 or the BLM-DNA2 ensemble, the 3′ ssDNA tails are coated by the RPA heterotrimer. In the central step of HR, BRCA2 with the help of BRCA1 and PALB2 delivers RAD51 monomers to ssDNA, resulting in RPA removal and RAD51 presynaptic filament formation required for strand invasion and homology search. Interestingly, in G1 phase, BRCA1-PALB2-BRCA2-RAD51 complex formation is impaired by proteasome-mediated degradation of PALB2 (7). Mechanistically, PALB2-interacting protein KEAP1 in complex with cullin-3-RBX1 ubiquitylate PALB2, thereby suppressing PALB2-BRCA1 (23). HR in somatic cells is mostly completed by synthesis-dependent strand annealing (SDSA), generating non-crossovers, although other outcomes are possible (24).

### Alternative DSB Repair Pathways

A-EJ is genetically distinct from Ku-dependent c-NHEJ and RAD51-dependent HR and requires the presence of microhomology (MH) regions (2–20 bp), which are exposed following MRN-CtIP-mediated resection [(25, 26); Figure 1]. Importantly, long-range resection impedes a-EJ and favors HR or SSA (27, 28). DNA polymerase theta (Polθ), a low-fidelity DNA polymerase-helicase, has been recently identified as key factor driving a-EJ by limiting RAD51 nucleation onto ssDNA (29–31). The Polθ-helicase domain displaces RPA from ssDNA tails, whereas the Polθ-polymerase domain promotes their synapsis, thereby facilitating MH-mediated annealing and subsequent gap filling (32, 33). The essential ligation step during a-EJ is performed by the DNA ligase IIIα-XRCC1 complex (26). Contrary to a-EJ, SSA requires more extensive DNA end resection followed by RAD52-mediated annealing of homologous tandem repeat sequences (>20 bp) [(34); Figure 1]. Whether a-EJ and SSA serve primarily as backup pathways in mammalian cells deficient in either c-NHEJ or HR, or are favored at specific genomic loci still remains to be established (35).

## DSB Repair Protein Dysfunction in Cancer

Only a minor number of human cancers are associated with downregulation or alterations of core c-NHEJ genes (36). Rare mutations in *LIG4* (encoding DNA ligase IV), *XLF, DCLRE1C* (encoding Artemis) or *PRKDC* (encoding DNA-PKcs) have been identified in a radiosensitive sub-class of patients with severe combined immunodeficiency (SCID) and can predispose to cancer (37, 38). As c-NHEJ is the predominant DSB repair pathway in human cells, complete loss-of-function is likely to drive cell death due to an unreasonably high DSB burden (36). Elevated DNA-PKcs levels were implicated in the progression of various types of tumors such as prostate cancer and melanoma (36). Noteworthy, *PRKDC* is with 2.1% the sixth most frequently mutated DNA repair gene in all cancers and considered a potential oncogene, exhibiting frequent copy number gains (39).

A comprehensive analysis of somatic DDR gene alterations delineates HR as the most frequently altered DNA repair pathway across 33 cancer types, most notably ovarian cancer (40). Mutational signatures associated with robust HR deficiency (HRD) primarily included alterations affecting *BRCA1, BRCA2*, two canonical RAD51 paralog genes (*RAD51B, RAD51C*), *BLM*, and *RAD50* (40). Large-scale molecular profiling of solid tumor samples across 21 cancer lineages detected pathogenic HR gene mutations with an overall frequency of 17.4%. Here, again, *BRCA2* (3%) and *BRCA1* (2.8%) were the most commonly mutated bona fide HR genes and predominantly seen in ovarian and breast cancers (41). Heterozygous germline mutations in *BRCA1* and *BRCA2* are responsible for the majority of hereditary breast and ovarian cancer (HBOC) syndrome patients. However, only ~20–25% of HBOC families have *BRCA* mutations and other low-to-moderate penetrance HBOC susceptibility genes involved in HR have been identified, including *BRIP1, RAD51C*, and *PALB2* (42). Moreover, revisiting whole-exome sequencing datasets of non-BRCA1/2 familial breast cancer patients confirmed the existence of likely pathogenic germline variants in *MRE11A, RAD50*, and *NBN*, encoding components of the MRN complex (43, 44). Lord and Ashworth have coined the term “BRCAness” to denote HRD tumors that share molecular features of *BRCA1/2*-mutant tumors and are therefore expected to effectively respond to the same treatment modalities (45). Remarkably, however, a recent study indicated that most somatic *BRCA1/2* alterations in non-BRCA associated cancer types may be incidental findings unrelated to tumor pathogenesis, rendering them therapeutically irrelevant (46). In contrast to the situation encountered for *BRCA1/2*, no inactivating mutations of *RAD51* have been reported in tumors. Paradoxically, *RAD51* is frequently found overexpressed and has been associated with poor prognosis in patients with solid malignancies, thus potentially acting as a driver of aberrant HR (47).

Similarly, elevated MRN expression has been correlated with tumor progression and poor survival in patients with rectal and gastric carcinomas and prostate cancer (48–50). However, with the exception of a positive relationship between MRN deficiency and microsatellite instable (MSI) colorectal cancers, large scale studies will be required to substantiate its relevance in clinical settings (51). Like MRN, CtIP also has rather oncogenic potential at the cellular level, most likely by facilitating a-EJ-dependent chromosomal instability (52–54). Accordingly, mice heterozygous for a null *Ctip* allele did not display increased tumor susceptibility, meanwhile CtIP inactivation suppressed mammary tumorigenesis caused by p53 deficiency (55). Although still far from being fully characterized, a-EJ is intrinsically mutagenic, typically generating deletions at the repair junction, and suggested to be a major driving force of genomic instability in human cancers (56–58). In particular, a-EJ reliant on Polθ, also referred to as theta mediated end joining (TMEJ, see Figure 1), has emerged as a distinct DSB repair pathway acting predominantly in HRD tumors or on breaks incompatible with c-NHEJ and HR (59). Consistently, depletion of BRCA1/2 resulted in increased usage of TMEJ using reporter assays in human cells (25). Elevated *POLQ* (encoding for Polθ) expression has been described in numerous cancer types, including breast and ovarian cancer (29, 59–61). Overall, CtIP and Polθ may drive tumorigenesis through a-EJ in defined biological contexts and therefore represent promising therapeutic targets.

## DSB Repair Proteins as Drug Targets

As outlined above, DSB repair constitutes an Achilles' heel of cancer cells and there is a continuous search for compounds specifically targeting DSB repair components to exploit this key vulnerability.

### Combinatorial Treatment Regimens Involving DSB Repair Inhibitors

DNA repair targeted therapy was first considered most beneficial in combination with conventional DNA-damaging agents (62, 63). In recent years, mainly thanks to the development of PARPi, additional treatment strategies including DDR inhibitor combinations have been implemented in clinical trials (3, 64, 65). Furthermore, DDR-targeting drugs were found to enhance the effectiveness of immunotherapy by fostering increased immunogenic surveillance and restricted tumor growth (66, 67). An elevated mutation load was shown to increase neoantigen levels in cancer cells thereby promoting tumor immunogenicity (68). Here, we will mainly focus on available DSB repair pathway inhibitors and their synergistic effect in combination with standard chemo- or radiotherapy. Moreover, existing PARPi-based combination strategies will also be highlighted.

#### Pharmacological Targeting of c-NHEJ

Restraining c-NHEJ capacity has been primarily achieved by targeting DNA-PK (Figure 1). Conceptually, compounds blocking c-NHEJ are thought of as being most effective when used in combination with radiation therapy, as c-NHEJ is taking care of roughly 80% of IR-induced DSBs (69). Whereas, numerous DNA-PKcs small-molecule inhibitors (DNA-PKi) have been developed over the last 20 years, only one specific agent is known to target the Ku heterodimer (70). Weterings et al. identified a compound interfering with the binding of Ku to DNA and sensitizing human cell lines to IR (71). Similarly, the majority of DNA-PKi displayed synergistic effects with IR and chemotherapeutics including etoposide and cisplatin (72). For example, VX-984 induced radiosensitivity of glioblastoma cells grown as orthotopic xenografts (73), whereas combination of the DNA-PKi KU-0060648 with ATR inhibitor AZD6738 potentiated radiosensitization of head and neck squamous cell carcinoma cell lines (74). The most potent DNA-PKi (M3814, CC-115 and CC-122) are currently being investigated in several clinical trials (72). Of particular interest, a dose escalation phase I clinical trial combines M3814 with Avelumab (NCT03724890), a human monoclonal antibody targeting the protein programmed death-ligand 1 (PD-L1). Remarkably, CC-115, a dual inhibitor targeting DNA-PK and the structurally related mammalian target of rapamycin kinase (TORK), was shown to induce caspase-dependent cell death in primary chronic lymphocytic leukemia (CLL) cells and to be clinically effective in CLL patients with an *ATM* mutation (75, 76). However, it remains an open question of whether DNA-PKi act solely by impairing DSB repair, as other cellular functions of DNA-PKcs have been reported, including cell cycle progression, transcription and telomere maintenance (70).

#### Pharmacological Targeting of HR

MRE11 harbors endo- and exonuclease activity essential for DNA end resection, thereby channeling DSBs into homology-directed repair pathways (22). A forward chemical genetic screen identified mirin as the first MRE11 inhibitor targeting its exonuclease activity and preventing ATM activation (77). In addition, structure-guided nuclease-specific MRE11 inhibitors revealed that endonuclease inhibition promotes c-NHEJ in lieu of HR, whereas exonuclease inhibition caused a more profound DSB repair defect (78, 79). CtIP's role in DSB resection has been mostly attributed to its interaction with and stimulation of MRE11, although intrinsic CtIP endonuclease activities have also been demonstrated (80–83). Intriguingly, CtIP-specific inhibitors have not been reported yet. However, inhibition of Bromodomain-containing protein 4 (BRD4) was found to induce an HRD signature by decreasing transcriptional activity of the *CtIP* promoter and enhancer (84). Reduced CtIP protein levels correlated with increased PARPi sensitivity, potentially qualifying CtIP as a predictive marker for PARPi response. Consistently, different BRD4 inhibitors (e.g., JQ1 and AZD5153) sensitized a broad range of tumor types to PARPi in multiple *in vitro* and *in vivo* models (85).

BRCA1 and BRCA2 represent challenging targets for structure-based drug discovery, as they are both large proteins made up of short, functional domains, serving as hubs for multiple protein-protein interactions, interspersed by long, intrinsically disordered linkers (86). In this regard, Pessetto et al. identified a cell permeable peptide ablating phosphoprotein binding by the BRCA1 tandem BRCT domains and enhancing PARPi sensitivity of cancer cells (87). Similarly, a BRCA2-mimetic cell-penetrating peptide disrupting BRCA2-RAD51 interaction conferred PARPi sensitivity in cancer cell lines (88). Small molecules selectively targeting BRCA1's ubiquitin ligase activity, which is mediated by the N-terminal RING domain and required for efficient DSB resection (89), might also offer a valid alternative to inhibit HR.

Chemical inhibitors of RAD51 (e.g., B02, IBR2, RI-1/2) have been reported to either interfere with RAD51 oligomerization, filament formation or DNA binding, and, ultimately, to induce HR deficiency [(78, 90–94); Figure 1]. Triple combination of B02, the PARPi veliparib and a p38 MAPkinase inhibitor (LY2228820) significantly reduced primary tumor growth in an orthotopic triple negative breast cancer (TNBC) mammary xenograft model (95). Similarly, cancer cell proliferation in a breast cancer xenograft model and in a chronic myelogenous leukemia model bearing the BCR-ABL^T315I^ mutation was significantly slowed upon IBR2 treatment (94). RI-1 potentiated the effect of the alkylating agent Iomustine on a glioma xenograft model, reduced growth of cervical cancer xenografts and hindered TNBC growth *in vivo* when combined with veliparib (96–98). Based on these preclinical findings, RAD51i were proposed as potential candidates for a novel class of broad-spectrum therapeutics for difficult-to-treat cancers. Interestingly, Cyteir Therapeutics is currently recruiting patients for a phase 1/2 study with CYT-0851, an oral RAD51i designed to reduce the ability of RAD51 to migrate to and from sites of excessive DNA damage (NCT03997968). In addition to direct RAD51 inhibition, inactivation of RAD51 can also be achieved by indirect mechanisms, including tyrosine kinase inhibitors (93). For example, it was recently reported that cediranib (AZD-2171), a potent inhibitor of vascular endothelial growth factor (VEGF) tyrosine kinases, constrains HR through transcriptional repression of *RAD51* and *BRCA1/2* (99). Accordingly, combination of the PARPi olaparib with cediranib showed superior progression-free and overall survival outcomes in relapsed ovarian cancer patients without documented *BRCA1/2* mutations (100).

Even though drugs inhibiting c-NHEJ or HR have proven highly effective in combinatorial treatment strategies, they usually lack tumor specificity and receiving patients often suffer from toxic side effects, resulting in a narrow therapeutic window. Nowadays, SL-based strategies provide a more promising approach for therapeutic interventions, particularly in patients with HRD.

### Exploiting Synthetic Lethality in HR-Defective Tumors

**Figure 2:**
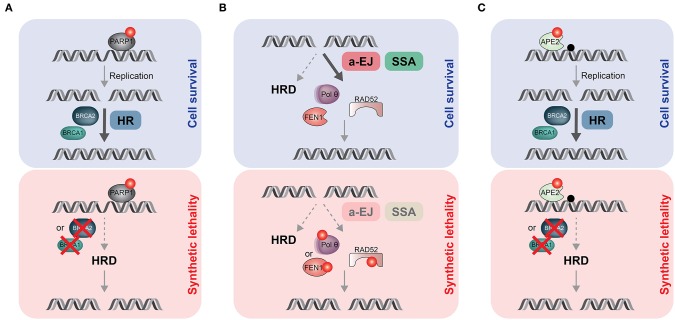
Synthetic lethal (SL) strategies to target HR-deficient (HRD) tumors. **(A)** PARPi (red dot) trap PARP1 proteins on endogenous DNA lesions, including single-strand breaks or gaps. If not removed timely, trapped PARP1 blocks the replication machinery, leading to one-ended DSBs that need to be repaired by HR. In HRD tumors such as *BRCA1*- or *BRCA2*-mutated cancers, DSBs persist and accumulate, ultimately causing cell death due to SL. **(B)** HRD tumors are addicted to alternative, mutagenic DSB repair process (a-EJ and SSA) to sustain proliferation. Targeting Polθ (or FEN1) or RAD52 by small molecule inhibitors (red dots) would eliminate a-EJ or SSA, respectively, resulting in SL. **(C)** Apurinic/apyrimidinic (AP) sites (black dot) are one of the most frequent spontaneous lesions in DNA. If not timely removed by APE2, they have the potential to block DNA replication. Consequently, APE2 inhibition (red dot) would lead to massive accumulation of AP sites associated with increased rates of fork collapse and DSB formation. In healthy cells, HR can deal with those DSBs, promoting cell survival. In contrast, treating HRD tumors with APE2i would cause DSB-induced cell death due to SL.

The most popular synthetic lethal interaction (SLI) exploited in cancer therapy is the one between *BRCA* and *PARP1* genes (101, 102). Catalytic inhibition of PARP1 “traps” PARP1 molecules on damaged DNA, resulting in replication fork collapse and DSB formation. In combination with HRD, due to *BRCA1/2* loss, PARP trapping leads to persistent accumulation of DSBs, inducing cell cycle arrest and apoptosis (Figure 2A). In two landmark studies, pharmacological targeting of PARP1 with the orally active PARPi olaparib showed a favorable therapeutic index in homozygous BRCA-mutated breast or ovarian cancer (103, 104). There are currently six small-molecule PARPi available in the clinic, four of them (olaparib, rucaparib, niraparib and talazoparib) have already obtained approval in different therapeutic settings (65). Despite this remarkable success story, resistance to PARPi remains a major problem in the clinic and an active area of research (105). Nonetheless, the identification of additional, cancer-specific SL gene pairs holds great promise in developing effective monotherapy regimens, as exemplified below (Figure 2B).

Two seminal studies from the Sfeir and D'Andrea laboratories established that HRD cancers display a pronounced dependency on TMEJ to limit the toxicity of DSBs [(29, 30); Figure 2B]. Moreover, the fact that Polθ is generally absent in normal cells but upregulated in many cancers makes it a highly desirable drug target (29). Consequently, two established precision oncology companies, Artios Pharma and Repare Therapeutics, have launched Polθ inhibitor programs with first-in-human clinical studies due to start soon. Furthermore, CRISPR-based genetic screens targeting 309 murine DDR genes identified 140 *Polq* SL genes, including many HR mediators, several c-NHEJ genes and key components of the 53BP1 anti-resection pathway (106). Notably, 30% of human breast cancers in the TCGA cohort were found to be likely deficient in one or more of the 140 *Polq* SL genes, significantly broadening the number of patients that may benefit from Polθ inhibition (106).

Another interesting SLI was repeatedly reported between *RAD52* and *BRCA1/2* [(107–111); Figure 2B]. Due to the multiple roles of RAD52 in genome maintenance pathways, the exact mechanism underlying the RAD52-BRCA SL remains to be fully understood (112). However, it has been reported that RAD52-dependent SSA acts as an important backup when direct protein-protein interactions in the BRCA1-PALB2-BRCA2 complex, required to channel resected DSBs down the HR path, are disrupted (113). In large agreement with this notion, RAD52 inhibitors exerted synergistic activity with PARPi against BRCA1-deficient tumor cells (114). Remarkably, combined disruption of *RAD52* and *POLQ* caused additive hypersensitivity to cisplatin, indicating distinct back-up roles in DSB repair and a potentially effective approach for SL therapeutic strategies (115). Several small-molecule RAD52 inhibitors have been developed, but none of them have been subjected to clinical trials (78).

Last but not least, genetic screens by the Elledge laboratory uncovered *FEN1* (encoding Flap endonuclease 1) and *APEX2* (encoding AP endonuclease 2, APE2) as SL genes in *BRCA1/2*-deficient backgrounds (116). They proposed that in the context of HRD, FEN1 may be responsible for the removal of Polθ-dependent 5′ flaps during TMEJ (Figures 1, 2B), while APE2 is mainly processing abasic sites at replication forks to avoid fork collapse and DSB formation [(116); Figure 2C].

Notably, acquired genomic instability due to HRD facilitates acquisition of mutations that could trigger therapy resistance (4). For instance, PARPi resistance mechanisms have mostly been linked to either reactivating *BRCA* mutations or DDR rewiring, thereby functionally restoring HR. In these cases, chemical inhibition of the reactivated HR pathway has been proposed to overcome PARPi resistance (117). Interestingly, numerous studies revealed that reversion mutations of *BRCA* genes display MH signatures that likely originate from error-prone DSB repair mechanisms such as a-EJ and SSA (118). Consequently, combined inhibition of PARP1 and Polθ (or RAD52) should prolong drug responses and prevent resistance acquisition (118). In addition, targeting alternative SLIs with HRD (Figure 2C) could be beneficial when PARPi resistance arises due to loss of PARP1 expression or activation (117).

Finally, it remains to be said that only few robust SLIs have been identified since the discovery of the SL between PARP inhibition and *BRCA1/2* loss of function in 2005 (119). Moreover, it has been argued that most SLIs display incomplete penetrance due to extensive molecular heterogeneity seen in tumors (120). Therefore, assessing the penetrance of SLIs will become an important aspect of future research.

## Conclusions

It has become increasingly evident that targeted inhibition of DSB repair proteins offers a wide range of possible applications in cancer treatment. Initially, combinatorial therapy of DSB repair inhibitors with DNA-damaging agents (e.g., IR or cisplatin) were considered most effective. Given that DSB repair deficiency results in increased tumor immunogenicity, the combination of selected DSB repair inhibitors with immunotherapy will very likely find its way into the clinic. In addition, the emerging concept of exploiting SL as anti-cancer therapy is expected to allow more selective and efficient tumor killing without the side-effects of conventional drugs. Importantly, sequential therapy with DNA repair inhibitors was found to be less toxic compared to simultaneous drug administration meanwhile retaining treatment efficacy (121, 122). Consequently, detailed evaluation of the drug administration timing is of vital interest to reduce cytotoxicity. In addition, the stratification of robust biomarkers and detection of mutational signatures will be highly critical to the implementation of SL but also combinatorial therapy regimens (123). Finally, DSBs are repaired by multifactorial pathways that are heavily connected. These interdependencies generate potentially druggable vulnerabilities but also opportunities for tumors to develop drug resistance. Thus, establishing potent inhibitors for each DSB repair pathway will create new treatment opportunities for a wide range of tumors.

## Author Contributions

All authors listed have made a substantial, direct and intellectual contribution to the work, and approved it for publication.

### Conflict of Interest

The authors declare that the research was conducted in the absence of any commercial or financial relationships that could be construed as a potential conflict of interest.
